# A look behind the scenes: COVID-19 impact on depression and perceived stress of UAE population

**DOI:** 10.1186/s43045-021-00115-7

**Published:** 2021-06-29

**Authors:** Zelal Kharaba, Sayer Al-Azzam, Ahmed Alhusban, Khawla Nuseir

**Affiliations:** 1 Department of Clinical Sciences, College of Pharmacy, Al Ain University, Abu Dhabi, 112612 United Arab Emirates; 2 Institute of Cellular Medicine, Faculty of Medical Sciences, Newcastle University, Newcastle upon Tyne, UK; 3 Department of Clinical Pharmacy, College of Pharmacy, Jordan University of Science and Technology, Ar-Ramtha, Jordan

**Keywords:** COVID-19, Coronavirus, UAE, Depression, Perceived stress, PHQ-9, PSS

## Abstract

**Background:**

The COVID-19 pandemic is taking many lives every day. It affects literally all aspects of life and changes humanity communication tools, mobility, lifestyle, and feasibly their level of perceived stress and depression. The present study aims to investigate the psychological health status in terms of perceived stress and depression among the UAE population in response to the COVID-19 pandemic. This is a cross-sectional questionnaire-based study in the UAE during the lockdown period of the COVID-19 pandemic. Validated assessment tools (PHQ-9 and PSS) were used to assess depression and perceived stress, respectively. Data were analyzed using categorical data and mean± SD for scores of perceived stress, and depression scales were calculated. The SPSS statistical software (version 24.0) was used for data analysis purposes.

**Results:**

Our findings revealed that the pandemic has significantly influenced the daily routine and psychological health. Depressive symptoms were prevalent in 47.8% of the participants. A concerned percent of participants around 84% were anxious. Age, gender, school attendance, and the impact of the pandemic on work performance were the major factors of developing depression and perceived stress symptoms.

**Conclusion:**

The COVID-19 pandemic has a considerable negative psychological impact on the public in the UAE. The importance of the current study also came from the fact that only very few or no study has holistically evaluated the psychological state of the public during the lockdown in the UAE. The figures that reflect the depression and the perceived stress level among the public during the lockdown also struck our attention. Almost half of the participants in this study suffered from depression. Also, approximately 85% of the same were anxious during the lockdown. These figures should not be overlooked when further psychological assessment studies are conducted.

## Background

Nowadays, people over the entire globe are facing the threat of the fifth pandemic after the 1918 flu pandemic. The world was shocked by the pattern of coronavirus disease-2019 (COVID-19) since the first report of Wuhan Municipal Health Commission, China, that showed a cluster of cases of pneumonia in the late of December 2019 [1]. Later on, and very aggressively, the viral transmission was accelerated and became a monster that affected more than 50 million person and cause the death for more than 1.2 million people in the first few months of 2020 [2]. The killing power and the deadly transmission from human to human in vast majority of cases especially in people with comorbidities have required most of the governments to take different actions and measures as an attempt to break the exponential line of incidence that lead to restrict human mobility and impose a burden on health care system and the economy.

In the United Arab Emirates (UAE), the first cases of COVID-19 that reached the Middle East were confirmed by a 4-member Chinese family who came from China to the UAE as tourists in the 16th of January 2020. The UAE’s Ministry of Health and Prevention (MOHAP) has adhered to the principles of transparency in dealing with these cases and ensured publicity. In the 3rd of March 2020, the MOHAP affirmed a total of 27 cases with positive COVID-19 test, and since that, the number of cases has started to increase dramatically [3]. Consequently, all governmental institutions have taken actions toward this crisis; for instance, the UAE’s Ministry of Education has applied the distance learning [4] and the economical movement has been limited; thereby, many employees had either distance working or unpaid annual leaves [5].

Historically, many countries employed a compulsory quarantine during many previous infectious crises as cholera, plague, and SARS [6, 7]. However, the worldwide lockdown in the current crisis is extraordinary since public quarantine is needed to limit the spread of the virus. On the other side, this situation has led to many problematic consequences on economical, behavioral, social, and educational life as well as psychological imbalance [8]. For instance, in Taiwan, the frontline nurses in SARS unit during the SARS epidemic were suffering from depressive symptoms compared to nurses in non-SARS units [9]. Likewise, many healthcare providers were living in unavoidable fear and stress during the COVID-19 crisis in different countries [10, 11].

In the UAE, a systematic quarantine and a night curfew for sterilization campaign over the whole emirates have been strictly implemented. The sterilization process has been completed in June 2020, and all residents were instructed to wear medical masks. Thus, the pandemic has changed deeply the conventional way of life in different perspectives and opens the door for many scientific questions that require answers. One important aspect—yet to be examined—is the impact of the pandemic on the psychological health status of people who feel depression and perceived stress. For these purposes, we conducted a cross-sectional study that allows us to grasp a deeper understanding of UAE community’s depression and perceived stress and to endorse coping strategies with the pandemic at national and international level.

## Methods

### Study design and sampling technique

This was a cross-sectional questionnaire-based study using a structured online survey conducted over a month period (May 14th to June 17th, 2020) on males and females across the UAE. The study population included males and females (n=209) who were >18 years and consented/agreed to participate in the study. Questionnaires required an average of 6 min to be completed. The study participants were informed about the study objectives, and the data were collected and treated with total confidentiality. Consents for participation were obtained from of all study participants before data collection. The study received the required ethical approval from the Research Ethics Committee at Al-Ain University (REC/AAU/B3/May2020) Abu-Dhabi, UAE, and from the institutional review board at Jordan University of Science and Technology (IB/JUST, May 2020) in Jordan-Irbid. Convenience sampling technique was used in this study.

### Participants

The following information was addressed: age, marital status, occupation, and education and other sociodemographic characteristics and data regarding personal experience and burden during the pandemic’s curfew. Participants were invited to fill an online survey through different social media applications using a research snowball method during the lockdown period in the UAE.

### Study tool, validation, and reliability testing of the study questionnaires

An assessment of psychological health was carried out using two validated tools, the Patient Health Questionnaire-9 (PHQ-9) for depression and the Perceived Stress Scale (PSS) for perceived stress.

The PHQ-9 is a 9-item self-reported questionnaire, and it is one of the most prevailing validated tools used by clinicians to assess the presence of depressive symptoms and monitor the treatment response [12]. It has a 4-point Likert scale ranging from “not at all” to “nearly every day” with a maximum score of 27. The cutoff point is nine, where scores < 9 are labeled as no depressive symptoms, while scores > 9 affirmed the existence of depressive symptoms [13].

The PSS is a 10-item self-reported questionnaire, which mainly assesses the psychological conceptualization of stress and how the life events during the last months were perceived as uncontrollable and unpredictable. It has a 5-point Likert scale ranging from “never” to “very often” with a maximum score of 40. Higher scores reflect high levels of perceived stress [14].

### Inclusion and exclusion criteria

Males and females aged >18 years, live permanently in the UAE, and adhered strictly to the lockdown regulation directed by UAE government were included in the study. The exclusion criteria were living in the UAE occasionally (less than 1 year), participants with history of mental disorders, and those who refused to participate.

### Data collection

The study questionnaire was administered through online survey with participants who met the eligibility criteria and agreed to participate. The questionnaire distribution was carried out by the research team, comprising two research associates who were given one lecture on the topic and one training workshop on finalizing and completing the study survey; all were delivered by the principal investigator. Research associates approached eligible participants using different online/social media platforms: WhatsApp, Facebook, Instagram, emails, and others. Participants were firstly briefed about the study purposes and the estimated time needed to complete the survey at the beginning of the survey. Furthermore, the participants were informed about the anonymity and confidentiality of the applied policy.

### Statistical analysis

Descriptive analysis of variables was calculated as frequency and percentage for categorical data and as mean ± SD for scores of perceived stress and depression scales. The SPSS statistical software (version 24.0) was used for data analysis. Significance was considered at a P-value of ≤ 0.05, and all tests were 2-tailed t-tests. One-way ANOVA and independent t-tests were performed to compare the variables’ categories. Multivariate logistic and linear regression were used to investigate the association between predictors and outcomes and were represented as odds ratios (OR) and 95.0% confidence intervals (CIs).

## Results

### Sociodemographic characteristics of participants

This study was conducted on 209 participants with an average age of 30.9 ± 9.7 years old. Majority of the study participants were females (75.6%, n= 158), and more than half hold bachelor’s degrees (57.4%, n= 120). Participants were almost equally distributed between medical and non-medical sectors. About 17% (17.2%, n= 36) of the participants reported kind of exposure to COVID-19. About 35.9% (n= 75) of them were in a complete curfew, and majority spent > 8 weeks in the lockdown (68.4%, n= 143). Most of the participants have worked from home (62.2%, n= 130), and around 34.4% (n= 72) reported a negative consequence on their monthly income. Details of the sociodemographics are presented in Table 1.

**Table 1:** Baseline characteristics of the participants

Variables	Mean ± SD
**Age**	30.9 ± 9.7
**Variables**	**N (%)**
**Age categories**	
18–20	27 (12.9%)
21–40	152 (72.7%)
41–60	28 (13.4%)
>60	1 (0.5%)
**Gender**	
Male	51 (24.4%)
Female	158 (75.6%)
**Marital status**	
Single	100 (47.8%)
Married	104 (49.8%)
Divorced	5 (2.4%)
**Employment**	
Governmental	63 (30.1%)
Private	104 (49.8%)
Self-employed	14 (6.7%)
Unemployed	26 (12.4%)
**Educational level**	
Less than high school	9 (4.3%)
High school	31 (14.8%)
Bachelor	120 (57.4%)
Master of Science	37 (17.7%)
Ph.D.	12 (5.7%)
**School attendance**	
Not a student	145 (69.4%)
High school	15 (7.2%)
Undergraduate	35 (16.7%)
Graduate	14 (6.7%)
**Occupation**	
Medical	102 (48.8%)
Non-medical	103 (49.3%)
**Contact with COVID-19 patients**	
Yes	36 (17.2%)
No	151 (72.2%)
Not sure	22 (10.5%)
**Curfew type**	
Complete	75 (35.9%)
Partial	133 (63.6%)
None	1 (0.5%)
**Duration of curfew**	
< 1 week	4 (1.9%)
2–3 weeks	12 (5.7%)
4–8 weeks	49 (23.4%)
>8 weeks	143 (68.4%)
**Monthly income negatively affected**	
Yes	72 (34.4%)
No	108 (51.7%)
Not sure	28 (13.4%)
**Worked from home**	
Yes	130 (62.2%)
No	79 (37.8%)
**Work performance**	
Negative	119 (56.9%)
Positive	16 (7.7%)
No effect	73 (34.9%)

### Psychological health assessment

Table 2 presents means± SD scores of PHQ-9 and PSS and the influence of demographics on these scores. Depressive symptoms and perceived stress were highly prevalent among young adults (18–20 years old) with a PHQ-9 score of 13.4 ± 7.4 (p= 0.0001) and PSS score of 20.7 ± 4.2 (p= 0.001). Females were more vulnerable to be anxious compared to males (p= 0.001) with a PSS score of 19.2 ± 4.1. Participants at high school level showed a higher risk of developing depression (15.9 ± 7.1, p= 0.003) and perceived stress (22.6 ± 2.9, p= 0.001) compared to other levels of education. The fear of negative impact on business was among the main triggers for depression and perceived stress when the depression score hits 12.7 ± 6.7 (p<0.001) and 19.8 ± 4.1 (p<0.001) respectively with significant statistical difference compared to those unaffected.

**Table 2:** Target symptoms stratified according to participant’s baseline characteristics

Variables	Average depression score ± SD	P-value	Average anxiety score ± SD	P-value
**Total**	10.5 ± 6.6		18.7 ± 4.2	-
**Age categories**		**0.009**		**0.001**
18–20	13.4 ± 7.4		20.7 ± 4.2	
21–40	10.5 ± 6.2		18.8 ± 4.1	
41–60	7.7 ± 6.9		16.3 ± 3.1	
>60	4.0 ± 0.0		13.0 ± 0.0	
**Gender**		0.681		**0.001**
Male	10.2 ± 6.2		17.0 ± 3.9	
Female	10.6 ± 6.7		19.2 ± 4.1	
**Marital status**		0.11		0.11
Single	9.9 ± 6.9		18.3 ± 4.2	
Married	11.3 ± 6.1		19.2 ± 4.1	
Divorced	6.2 ± 7.4		16.0 ± 2.9	
**Employment**		0.48		0.49
Governmental	10.7 ± 7.0		18.4 ± 4.2	
Private	10.2 ± 6.6		18.7 ± 4.1	
Self-employed	13.0 ± 6.6		20.3 ± 3.8	
Unemployed	9.8 ± 5.9		18.5 ± 4.7	
**Educational level**		0.36		**0.01**
Less than high school	12.2 ± 7.1		20.3 ± 3.7	
High school	10.8 ± 7.5		19.1 ± 4.8	
Bachelor	11.0 ± 6.6		19.0 ± 4.0	
Master of Science	8.8 ± 6.0		17.8 ± 4.0	
Ph.D.	9.0 ± 4.8		15.2 ± 3.1	
**School attendance**		**0.003**		**0.001**
Not a student	9.9 ± 6.6		18.2 ± 4.2	
High School	15.9 ± 7.1		22.6 ± 2.9	
Undergraduate	11.5 ± 6.0		18.8 ± 4.4	
Graduate	8.5 ± 4.4		18.8 ± 2.3	
**Occupation**		**0.002**		0.60
Medical	10.4 ± 5.7		18.8 ± 4.0	
Non-medical	10.8 ± 7.3		18.8 ± 4.3	
**Contact with COVID-19 patients**		0.96		0.50
Yes	10.7 ± 7.2		19.1 ± 3.6	
No	10.4 ± 6.4		18.5 ± 4.2	
Not sure	10.7 ± 7.0		19.4 ± 4.5	
**Curfew type**		0.19		**0.03**
Complete	11.6 ± 6.5		19.5 ± 4.1	
Partial	10.0 ± 6.6		18.1 ± 4.1	
None	11.0 ± 0.0		24.0 ± 0.0	
**Duration of curfew**		0.13		0.11
< 1 week	11.8 ± 9.3		21.0 ± 4.7	
2–3 weeks	7.8 ± 5.0		18.7 ± 3.8	
4–8 weeks	9.1 ± 6.0		17.5 ± 4.3	
>8 weeks	11.2 ± 6.8		19.0 ± 4.1	
**Monthly income negatively affected**		0.25		0.34
Yes	9.5 ± 6.2		18.2 ± 4.4	
No	11.0 ± 6.9		18.9 ± 4.0	
Not sure	11.5 ± 5.9		19.4 ± 3.9	
**Worked from home**		0.90		0.92
Yes	10.1 ± 6.6		18.3 ± 4.2	
No	11.1 ± 6.6		19.3 ± 4.0	
**Work performance**		**<0.001**		**<0.001**
Negative	12.7 ± 6.7		19.8 ± 4.1	
Positive	8.1 ± 6.3		17.3 ± 3.7	
No effect	7.6 ± 4.9		17.1 ± 3.8	

Total, n (%), 100 (47.8%) had depressive symptoms and 177 (84.7%) were anxious

### Predictors of perceived stress and depression

Table 3 shows multivariate logistic regression analysis, which reveals that school attendance, working from home, and working status are independent risk factors for developing depressive symptoms. Again, high school students showed higher risk of developing depression (OR=3.72; 95% CI 1.02–13.58, p= 0.047) which supports our finding of the mean and SD. Furthermore, participants working from home were more susceptible to develop depression compared to those not working at home (OR=0.38; 95% CI 0.19–0.75, p= 0.005).

**Table 3:** Multivariate logistic regression analysis of depressive symptoms

Total, n (%)		
**Variables**	**OR (95% CI)**	**P-value**
**School attendance**	-	**0.041**
Not a student	1.48 (0.65–3.35)	0.35
High school	3.72 (1.02–13.58)	0.047
Undergraduate	0.27 (0.07–1.03)	0.056
Graduate	-	-
**Worked from home**	-	**0.005**
Yes	0.38 (0.19–0.75)	0.005
No	-	-
**Work performance**	-	**<0.001**
Negative	0.39 (0.12–1.25)	<0.001
Positive	0.23 (0.12–0.46)	0.11
No effect	-	-

Additionally, participants whom businesses and income were under the burden of the pandemic were at higher risk to develop depression (OR=0.39; 95% CI 0.12–1.25, p= <0.001).

Table 4 presents the linear regression analysis of perceived stress symptoms, which explained the variance of 35.4% (F (6, 1497) = 155.49, p <0.0001). Results revealed that age, gender, presence of depressive symptoms, and work status were the leading factors affecting the perceived stress symptoms.

**Table 4:** Linear regression model for perceived anxiety symptoms

Variables	Standardized coefficient beta	P-value
**Depression**	0.424	<0.0001
**Age categories**	−0.143	0.02
**Gender**	0.216	<0.0001
**Work performance**	−0.201	0.001

Adjusted R square 0.354; F (6, 1497) = 155.49; P-value= <0.0001

## Discussion

In this manuscript, we discuss the impact of COVID-19 crisis on the psychological health of publics in the UAE. To the best of our knowledge, this is the first study in this area that assesses COVID-19’s impact on mental health as depression and perceived stress among publics in the UAE.

In response to this crisis, the curfew and disruption of usual daily life have affected the individuals’ psychological health status. Assessment of psychological health in most of the recent studies have focused on the frontliners (healthcare providers) [15, 16] with minimal concern about the public, especially in the UAE [17]. Thereby, our study aims to evaluate the psychological health status in terms of perceived stress and depression among UAE population in response to this pandemic and to investigate the most affected groups of people by this crisis.

Our data revealed that COVID-19 has a substantial effect on the public mental health in the UAE. Depressive symptoms were prevalent among 47.8% of the participants with an average score of 10.5 ± 6.6. Likewise, the average perceived stress score was 18.7 ± 4.2. Age, gender, work performance, and school attendance were major predictors of both depression and perceived stress.

The emergent spread of COVID-19 over the world has caused a big fear and depression among a large portion of the world. Approximately, half of our study participants suffered from depressive symptoms (47.8%) during the pandemic. On the other hand, previous studies reported lower extent of depression among their populations. For instance, 31.0% of the studied population in Milan had depression [18]. Similarly, a study conducted in China represented 16.5% of the participants having depressive symptoms. In India, even fewer prevalence of depression (10.5%) was reported during this pandemic [19]. According to our data, more than ¾ of the participants were anxious (84.7%) during the pandemic. While this high value has exceeded the reported values of perceived stress in previous studies, as in a Chinese study, which reported a prevalence of perceived stress among 35.1% of the participants [20]. Another previous study reported that 42.0% of their participants were anxious since this pandemic [18]. This variance in the prevalence of depressive symptoms can be attributed to many factors, such as the “hypochondriac concerns,” which means worry and fear of being infected during the pandemic or when the epidemic is out of control [21]. In addition, the time of conducting the study and type of the imposed lockdown are considerable explanations of this variance, where studies conducted early with no or partial lockdown showed less prevalence of depression compared to those conducted at the mid of the crisis and during a complete lockdown. This suggestion is further supported by our findings, which showed higher levels of depression among participants exposed to complete lockdown.

In the current study, both depression and perceived stress were significantly affected by many social biodata. For example, age, gender, school attendance, and work performance were directly affecting the prevalence of depressive symptoms and perceived stress. In accordance with China and Spain’s studies, our data showed that gender was a main predictor of developing depression, as females reported higher levels of perceived stress compared to males [22, 23]. Surprisingly, these results are supported by various concepts derived from previous studies. For instance, women are well-known of being the essential caregiver at home in majority of the families, so, when there was closure of schools and other facilities, a huge burden at home occurs over the women’s shoulders [22, 24]. In addition, when working women started to work from home during the lockdown, their ability to perform duties at home and achieve successful work was conflicted and reduced substantially [22]. However, further gender-based assessment after the COVID-19 crisis should be evaluated to find out its impact on the psychological health between both genders.

Furthermore, younger age participants (18–20) and high school students were more likely to be depressed and anxious compared to other age groups. This agrees with previous records, where younger participants showed worse psychological health during the COVID-19 crisis [18, 25]. This can be also justified by the prolonged lockdown and distance learning as well as equivocation about the examinations, which had a negative impact on the students particularly. This finding is consistent with the drastic change in youngers’ life compared to their parents’ life during this pandemic [26].

Additionally, our finding revealed that insecurity and work status are important socioeconomic factors for developing psychological burnout. Participants whose work performance was negatively affected during the pandemic had higher rates of depression and perceived stress. This can be clearly related to the economic crisis and inability to generate a good income following the lockdown, which was mainly seen among self-employee participants that were more anxious about their businesses [27]. Moreover, participants who worked from home during the lockdown showed more depressive symptoms. This interestingly can be attributed to the disturbance of the daily routine and alerting the important activities as well as to a big worry of losing their jobs. Our findings are further supported by previous study [22].

Conclusively, the current study demonstrated that COVID-19 has a substantial impact on public psychological health, especially among certain group of people as females, young age people, high school students, and those whose work performance was negatively affected after the pandemic. Further in-depth concern should be directed to assess the mental health among those groups to implement strategies in combating psychological impairments during this crisis.

### Study strengths and limitations

There is limited information about the existence of such studies in the region. The current study dealt with exploring the impact of COVID-19 on mental health of publics in the UAE. The present study has some limitations. Firstly, the majority of the participants were at young age and undergraduates. Nevertheless, the proportion of community, which was targeted in this study, was wide enough to include males and females from age of 18 and above, yet the community in the UAE is considered to be fresh and youth. Moreover, big percentage of the study participants were university students, and this could be due to the population demographics in the UAE as majority of the UAE population falls in the age group of 25 to 54 years old. Youth dependency ratio is 16.4% compared to elderly dependency ratio which is 1.3% [28]. Furthermore, our study sample size was relatively not large and not highly representative with regard to the size of the population but is considered representative from different angle as it included male and female participants with different age, educational background groups, and different emirates in the UAE.

The present study also had some other limitations: The results of this study were reliant on the correctness and honesty of the participants’ responses that might lead to information bias. In addition, it was conducted within a short duration after imposing the country lockdown. However, the research team aimed to assess the mental health in a relatively short period before the country can release the lockdown and announce return back to normal life.

## Conclusion

In the light of the present study, we can conclude the following:

1. The COVID-19 pandemic has a considerable negative psychological impact on public in the UAE. The importance of the current study also came from the fact that only very few or no study has holistically evaluated the psychological state of the public during the lockdown in the UAE.
2. The figures that reflect the depression and the perceived stress level among the public during the lockdown also struck our attention. Almost around half of the participants in this study suffered from the depression. Also, approximately of 85% of the same were anxious during the lockdown. These figures should not be overlooked when further psychological assessment studies are conducted.
3. The data suggested that community segments are perceiving and handling the fear of the pandemic differently. Females, young age people, and high school students were more vulnerable toward the lockdown than other segments.
4. After all, fear of job loss was also among the forcing factors that seem to increase perceived stress and depression patterns during the lockdown.

## Data Availability

Not applicable.

## Abbreviations

COVID-19: Coronavirus disease-2019
CIs: Confidence intervals
MOHAP: Ministry of Health and Prevention
OR: Odds ratios
PHQ-9: Patient Health Questionnaire-9
PSS: Perceived Stress Scale
UAE: United Arab Emirates
